# Knowledge mapping and current trends of global research on snoRNA in the field of cancer

**DOI:** 10.7150/jca.87196

**Published:** 2023-08-21

**Authors:** Runsen Xu, Lina Wang, Junhui Hou, Xia Wang, Yibing Wang, Kefeng Wang

**Affiliations:** 1 Department of Urology, Shengjing Hospital of China Medical University, Shenyang 110004, China.; 2 Department of Anesthesiology, Shengjing Hospital of China Medical University, Shenyang 110004, China.

**Keywords:** Bibliometric study, Small nucleolar RNA, Cancer, Molecular mechanism, Diagnosis, Prognosis

## Abstract

Cancer is a major health hazard for humans. Recent studies have indicated the involvement of small nucleolar RNAs (snoRNAs) in the occurrence and development of cancer and indicated its potential role as a diagnostic/prognostic marker and therapeutic target. The purpose of this study was to use the bibliometrics method to analyze the published literature on this subject. We collected articles pertaining to the field of snoRNA and cancer from the Web of Science Core Collection database. The data were analyzed to identify the research hotspots and frontiers. The number of articles in this field was low in the early period. Chu Liang and Montanaro Lorenzo were the most prolific authors on this subject, while Jiang and Feng were the most frequently cited authors. In China, three institutions published the most articles, namely Wuhan Univ, China Med Univ, and Guangxi Med Univ. The journal with the highest number of articles on this subject was Oncotarget. The country with the most published articles was China. Analysis of keywords and burst words indicated that early studies mainly focused on molecular mechanisms. Available evidence suggests the involvement of snoRNAs in the molecular mechanism of cancer development and their potential role as a diagnostic and prognostic biomarker.

## 1. Introduction

Cancer is a major threat to human health. More than 19 million new cancer cases were diagnosed worldwide in 2020 1. In 2022, cancer accounted for approximately 1,700 deaths per day in the United States 2, and the corresponding number in China was approximately 7,500 per day 3. Therefore, research on cancer is a key imperative.

Decades ago, small nucleolar RNA (snoRNA), a non-coding RNA of 60-300 nucleotides in length, was discovered in the nucleolus 4, 5. snoRNAs can be categorized as C/D box snoRNAs and H/ACA box snoRNAs 6. Earlier studies suggested the involvement of snoRNA in the modification of pre-rRNA and the processing of ribosomal RNA(rRNA) 7. The advances in genome sequencing technology have enabled the discovery of several other snoRNAs 8. snoRNAs have been found to be closely related to the pathogenesis, diagnosis, prognosis, and other aspects of cancers 9-11.

Bibliometrics is a novel interdisciplinary subject that entails the use of mathematical and statistical methods for quantitative analysis of research results. It can enable a better understanding of the research hotspots of a particular subject 12, 13. Bibliometrics has been widely used in various fields of biomedicine 14. In bibliometrics research, VOSviewer and CiteSpace have been widely used for visualization analysis with good results 15.

Although there are literature reviews in the field of snoRNAs and cancer, no systematic bibliometric analysis has been published. Therefore, systematic review and analysis of the existing research on this subject can provide useful insights. The purpose of this study was to use the bibliometrics method to analyze the published literature pertaining to the role of snoRNAs in cancer. Through visual analysis, we investigated the correlation between snoRNA and cancer. Our findings may provide useful information for future clinical or theoretical research.

## 2. Materials and methods

### 2.1. Data source and screening strategies

**Figure 1:**
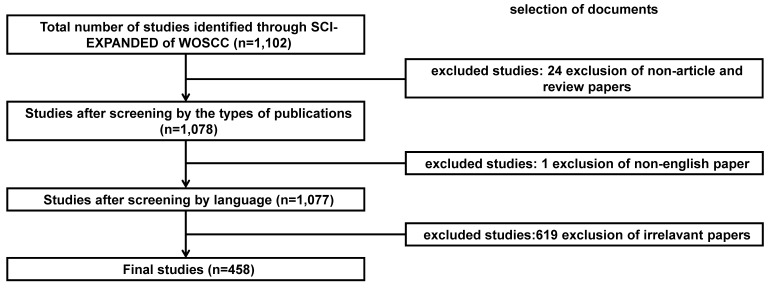
Flowchart for screening process of snoRNA's research in the field of cancer.

We searched the publications on snoRNAs in the field of cancer from January 1, 2000 to December 31, 2022, (from the publication time of the first article in this field to the start time of this study). Subsequently, Web of Science (WOS) Science Citation Index Expanded (SCIE) database was searched with the query of “(TS=(snoRNA*) OR TS=("small nucleolar RNA*")) AND (TS=((cancer) OR (carcinoma) OR ("malignant tumor") OR (malignanc*))”. The language of publication was limited to English and the types of publications were limited to “article” and “review”. On the initial screening of the retrieved papers, those with no snoRNA or any kind of cancer in the keywords were excluded, and papers with at least four kinds of small molecules including snoRNAs were retained; subsequently, articles in which snoRNA was not the main discussed topic were excluded. After careful review of the abstracts, papers that did not focus on the correlation between snoRNA and cancer were excluded. The literature retrieval strategy and the screening process are shown in **Figure 1**.

### 2.2. Data collection and analysis

The basic bibliometrics information for all publications was sourced from WOS core collection database. All selected documents were downloaded in plain text format with complete records and the cited references. WPS Office 3.9.6 Excel was used to process the annual variation function of publication volume. In addition, the top publications ranking was completed in WPS Excel.

VOSviewer 1.6.18 software was used for visual analysis, mapping correlations between authors, countries/regions, institutions/organizations, journals, keywords, and references. VOSviewer was used to display the clustering of each aspect of analysis and the analysis of data in the time dimension, which were respectively reflected in the network and overlay view. In both views, the size of the node represented the number of publications, and the thickness of the line represented the strength of the association between the two nodes. In the network view, nodes in the same cluster had the same specific color, and different colors represented different clusters. In the overlay view, the colors gradually changed over time, and nodes were given different colors depending on the time they represented.

CiteSpace 6.1.R3 was used to screen and generate burstiness of the keywords. We selected one year for each piece and eliminated the outburst keywords with strength lower than 2. The red area represented the outburst year of the keywords.

## 3. Results

### 3.1. Basic quantified information

A search of the WOS core collection database retrieved 458 articles related to snoRNAs in the field of cancer published between 2000 and 2022. The papers were written by 2,930 authors from 814 organizations across 50 countries and published in 235 journals, with a total of 23,322 references from 2,458 journals.

### 3.2. Analysis of annual change in publication volume

**Figure 2:**
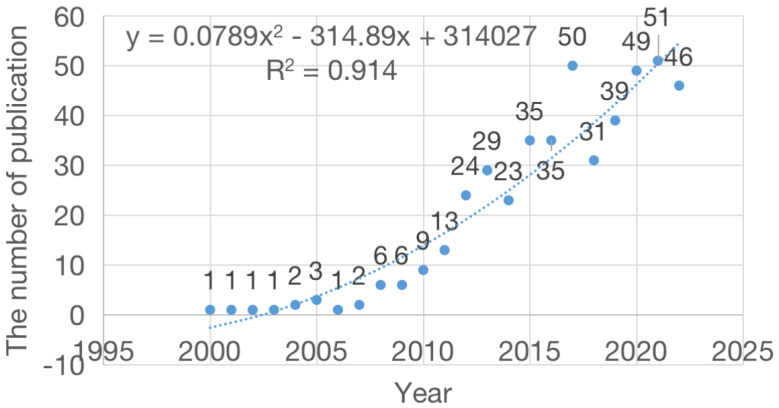
Annual publication regression curve of snoRNA's research in the field of cancer.

The collected data was sorted by time. A scatter plot of the annual number of published documents was prepared and a regression curve was drawn (**Figure 2**). Since 2008, the annual output showed an obvious growth trend, reaching a peak in 2021 with a total of 51 publications. Before 2008, the number of published papers was generally low, with a total of 12 papers in 8 years, accounting for 11.13% of the total articles. The average number of published papers over the past 10 years was 39. By fitting the data, we observed a statistically significant relationship between the year and the number of publications (r^2^ = 0.914). As shown by the curve, the number of published articles showed a rapidly increasing trend over successive years. In particular, in the past decade, the average annual number of published papers has stabilized at >20, indicating that an increasing number of researchers are working in this field. Based on the fitting curve, the estimated number of published papers in this field is projected to increase to approximately 63 in 2023.

### 3.3. Bibliometric analysis of the authors

**Figure 3:**
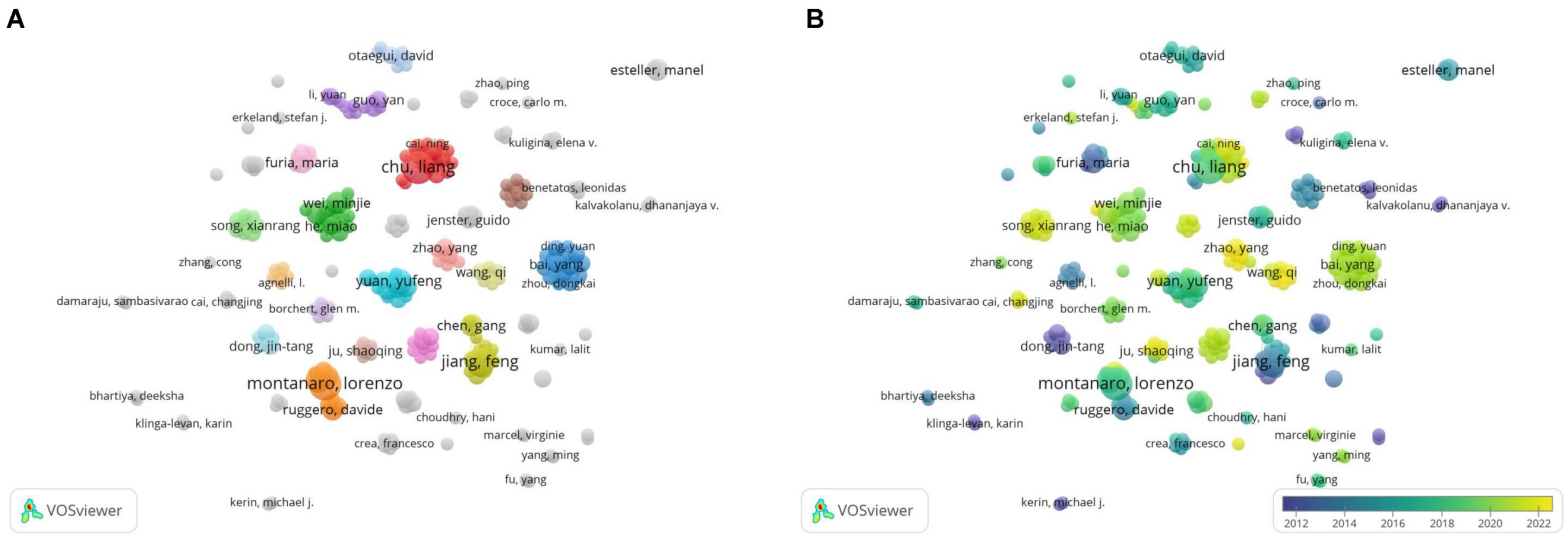
** A bibliometric analysis of authors co-authorship in the field of snoRNA and cancer.** (**A**). The network visualization map of authors collaboration in the field of snoRNA and cancer. (**B**). The overlay visualization map of authors collaboration in the field of snoRNA and cancer.

Analysis of the authors of the publications can help us identify the core authors and their main research directions in a certain field. According to Price's Law, the minimum number of publications by a core author is m=0.749×
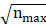
≈1.98. In the formula, 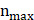 represents the author with the largest number of published papers (7 in this study). Therefore, authors who published more than two papers were identified as core authors in this field. A total of 256 researchers published 356 papers (77%), which was half (50%) of the standard proposed by Price's Law. The result suggests the formation of a stable group of collaborators in the field of research on snoRNA and cancer. **Figure 3A** displays the collaborative groups and the number of publications by each group and individual. **Figure 3B** shows the active period of each collaborative group in this field. The more yellow the color, the closer it is to today. The more purple the color, the further it was from today.

We further studied the highly productive authors. **Table 1** shows the five most productive authors. From 2000 to 2022, Chu Liang and Montanaro Lorenzo published the most papers (7 papers for both) and were cited 233 and 152 times, respectively. In 2019, Chu Liang published an article entitled “Small Nucleolar RNAs: Insight into Their Function in Cancer”, discussing several potential mechanisms by which snoRNA is associated with cancer. They mentioned that snoRNA may affect the occurrence and development of cancer by regulating p53 or phosphoinositide 3-kinase (PI3K)-AKT and Wnt/β-catenin pathways, or by affecting cancer stem cells 16. Montanaro Lorenzo, on the other hand, explored the link between snoRNA host genes and cancer 17. Jiang Feng, the author of the most cited paper, proposed the role of snoRNAs as a diagnostic biomarker in lung cancer, potentially opening the way for clinical use of snoRNAs 18, 19.

### 3.4. Bibliometric analysis of journals

The relevant journals in the research field of this paper largely pertained to the field of cancer, while a few had small molecular biology as their focus area. **Table 2** shows the top nine journals in terms of publication volume, among which Oncotarget, Plos one and the International Journal of Molecular Sciences published more than 12 articles. These three journals were open access, indicating an important role of open access journals in promoting this research field.

On citation analysis, the journal with the most citations per article was Oncogene, which focuses on genes and cancers, with an average of 112 citations. This indicated the high quality of the articles published in this journal and its wide readership among scholars in this field across the world.

### 3.5. Bibliometric analysis of countries/regions

**Figure 4:**
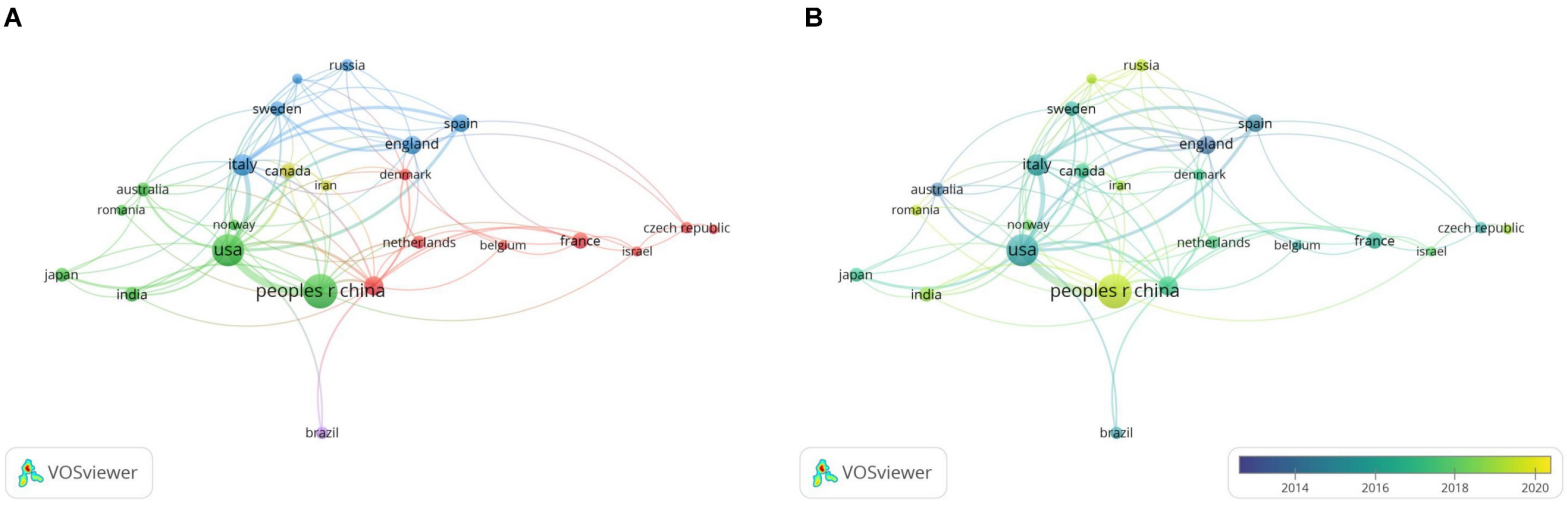
** A bibliometric analysis of countries/regions co-authorship in the field of snoRNA and cancer.** (**A**). The network visualization map of countries/regions collaboration in the field of snoRNA and cancer. (**B**). The overlay visualization map of countries/regions collaboration in the field of snoRNA and cancer.

To identify the countries that contributed the most in the research on snoRNAs in the field of cancer, we analyzed the number of publications in 49 countries. VOSviewer was used to visualize countries with 5 or more papers. The results are shown in **Figure 4**. The larger the circular node in the figure, the more publications the country has produced. The figure shows an uneven distribution of publishing countries in this field; the top effect was very significant, and most of the papers were authored by scholars from a few countries. The line of nodes represents the correlation strength. The thicker the node line, the more frequently the two countries collaborated in publishing articles. In **Figure 4A**, the node colors represent different clusters, and countries in the same cluster collaborated more closely. In **Figure 4B**, the node colors represent the active research time of the country. The figure shows that China was a relatively active country in this field of research in recent years.

**Table 3** shows the five most productive countries in this field. Chinese scholars published the most papers in the field, with a total of 147 papers, accounting for 32 % of the total papers in the field. Articles by Chinese authors were cited 3,259 times. It was followed by the United States, with 124 articles and 7,579 citations. England had the most citations per paper, with 27 papers cited 2489 times.

### 3.6. Bibliometric analysis of organizations

**Figure 5:**
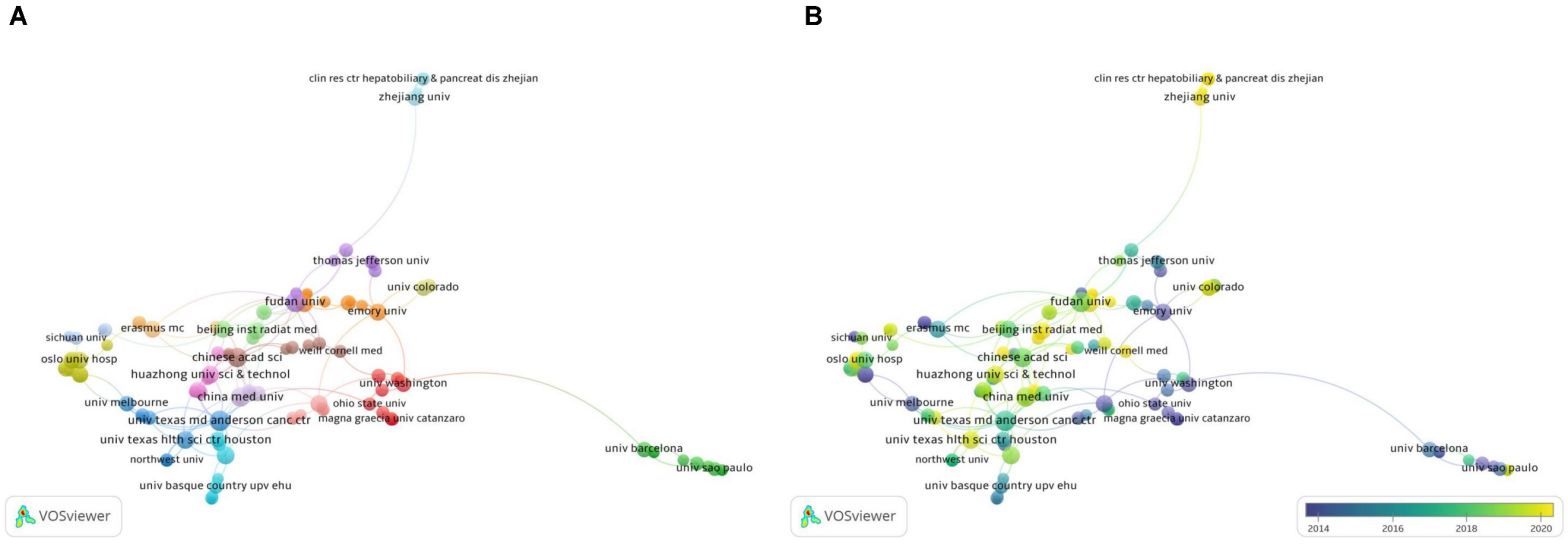
** A bibliometric analysis of organizations co-authorship in the field of snoRNA and cancer.** (**A**). The network visualization map of organizations collaboration in the field of snoRNA and cancer. (**B**). The overlay visualization map of organizations collaboration in the field of snoRNA and cancer.

Analysis of the research institutions can help characterize the input of the major research institutions in the field. This can facilitate better access to support and help for scholars and may facilitate collaboration between organizations. Through a visual analysis of organizations with more than four papers (**Figure 5**), we found that the collaboration between organizations was relatively extensive, but not fixed. Most organizations worked with each other no more than twice. The network map (**Figure 5A**) shows the organizations that collaborated more closely on research projects. The institutions with the most cooperation were Huazhong Uni Sci & Technol and Chinese Acad Sci. They collaborated in three projects, focusing on one particular snoRNA, SNORD126. The key output of their collaborative research was the finding that the activation of PI3K-AKT pathway can lead to the growth of liver cancer or colorectal cancer cells 20, as well as the proliferation of hepatocellular carcinoma (HCC) cells 21. It is worth noting that Chu Liang, the prolific author mentioned above, participated in and led these three research collaborations. This indicated that he played an important role in the collaboration between the two organizations and may further lead the development of this field in the future.

**Figure 5B** shows that several organizations in China have been active in publishing papers in recent years, which is also consistent with the results of the country visualization analysis above.

The top five institutions sorted in terms of publication volume are shown in **Table 4**. Wuhan Univ, China Med Univ, and Guangxi Med Univ published a total of 12 articles, demonstrating their leading position in this field. In September 2022, China Med Univ published an article demonstrating the promising role of SNORA38 as a prognostic marker in breast cancer, the most common malignant tumor in women worldwide 22.

### 3.7. Bibliometric analysis of keywords

#### 3.7.1. Co-occurrence analysis of keywords

**Figure 6:**
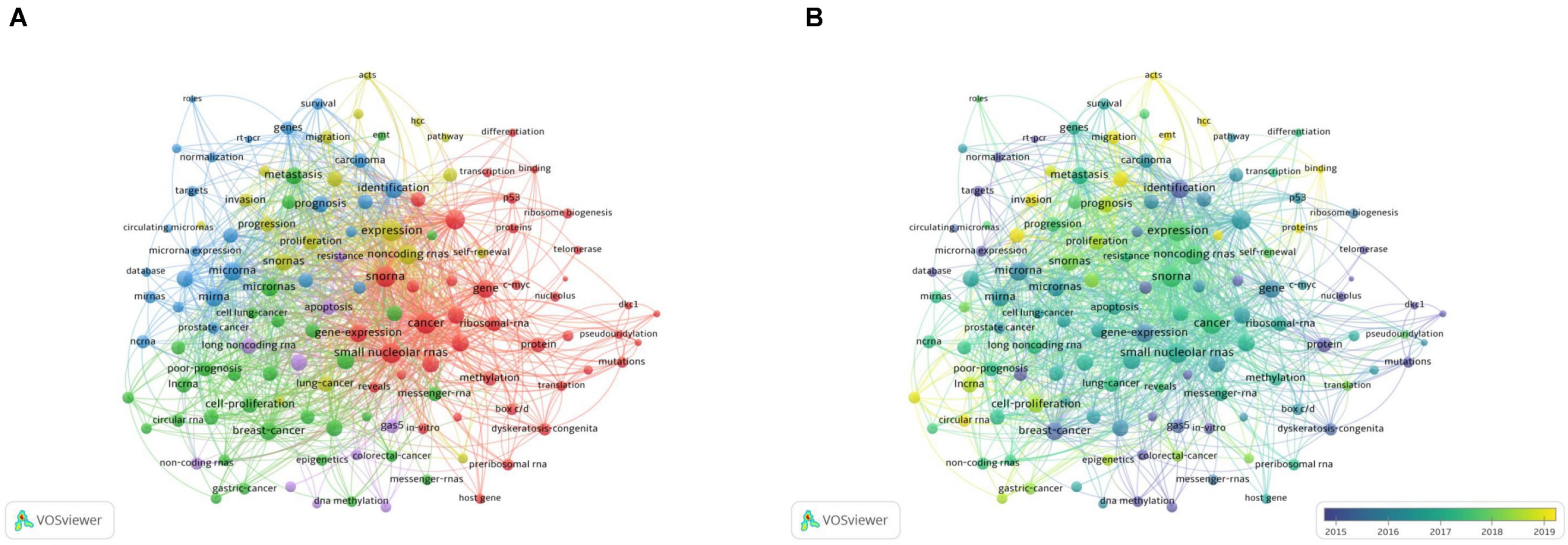

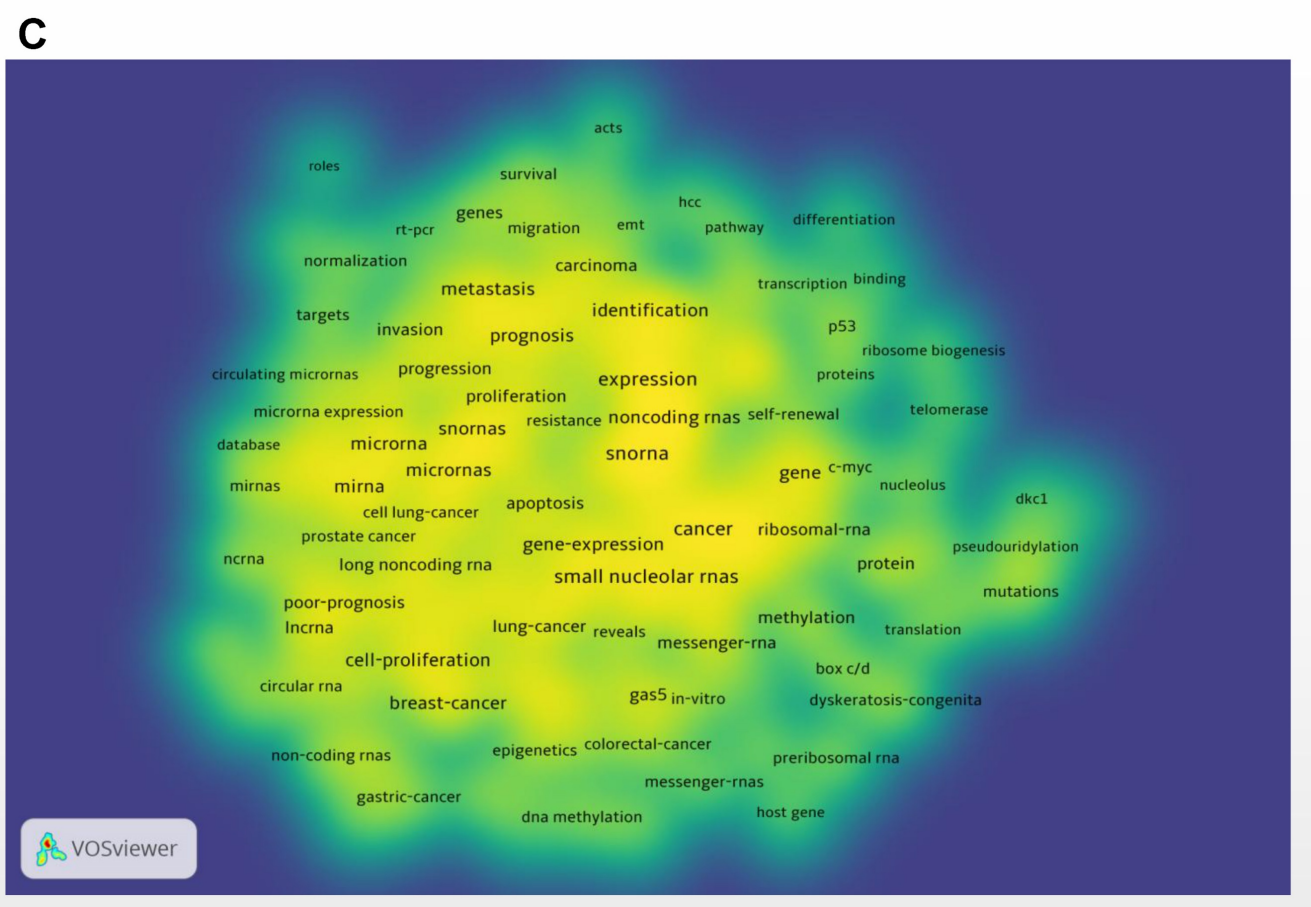
** A bibliometric analysis of keywords co-occurence in the field of snoRNA and cancer.** (**A**). The network visualization map of high frequency keywords in the field of snoRNA and cancer. (**B**). The overlay visualization map of high frequency keywords in the field of snoRNA and cancer. (**C**). The density visualization map of high frequency keywords in the field of snoRNA and cancer.

Keywords reflect the core of the article. Co-occurrence analysis of keywords can help identify the most intensive topic selection in a field. A keyword co-occurrence network of 458 selected articles was constructed using VOSviewer, and 127 keywords with a frequency of ≥ 8 were selected for visual analysis. In the network map (**Figure 6A**), the keywords were divided into four clusters. Cluster 1: 'biological molecule' (red color), including gene, protein, gene expression, and telomerase; cluster 2: 'related types of cancer' (green color), including breast cancer, prostate cancer, and gastric cancer; cluster 3: 'clinical application' (blue color), including identification, targets, biomarkers, survival, and prognosis. cluster 4: 'mechanism of oncogenesis' (yellow color): including expression, oncogene, progression, and pathway.

In addition, we overlayed a visualization map (**Figure 6B**) on the network map discussed above. The result indicated that basic research on molecular biology like cluster 1 was relatively popular before 2015, laying the foundation of the field. However, in recent years, more research has focused on clinical applications, which is a good sign. The theoretical research on the association of snoRNAs with cancer has gradually been translated into applications.

**Figure 6C** shows a density visualization map, which is similar in function to the network map, but more clearly shows the keywords that were of more interest in this research field.

#### 3.7.2. Burstiness of keywords

**Figure 7:**
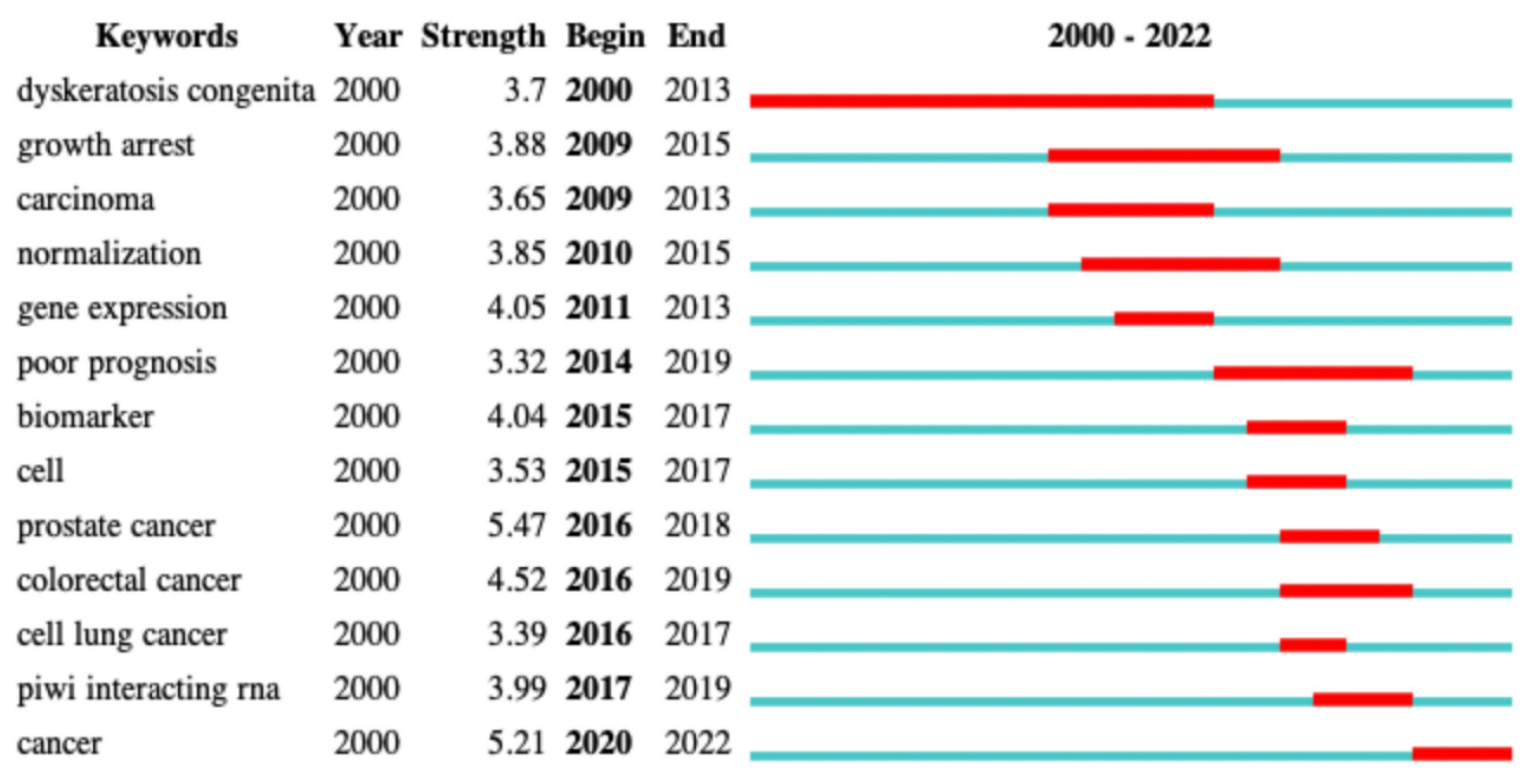
The burstness of keywords in the field of snoRNA and cancer.

Analysis of the burstiness of keywords can help identify the keywords that received the most attention from authors in a given period of time. This can help characterize the evolution of the most intensively researched topics. As shown in **Figure 7**, during a long period of time from 2000 to 2013, “dyskeratosis congenita” appeared in many articles as a high-frequency keyword. Dyskeratosis congenita was considered as a high-risk factor for some cancers 23-25. snoRNAs have been suggested to play an auxiliary role in the biological function of dyskerin. The mutation of dyskerin can cause dyskeratosis congenita and cancer 26. Therefore, dyskeratosis congenita broke out during that period. The subsequent keywords were mainly various cancers, such as prostate cancer, colorectal cancer, and cell lung cancer. Further research in this field has gradually unraveled the correlation of snoRNAs with various cancers, inducing a shift in the research direction from theoretical to clinical research.

### 3.8. Bibliometric analysis of Co-citation

**Figure 8:**
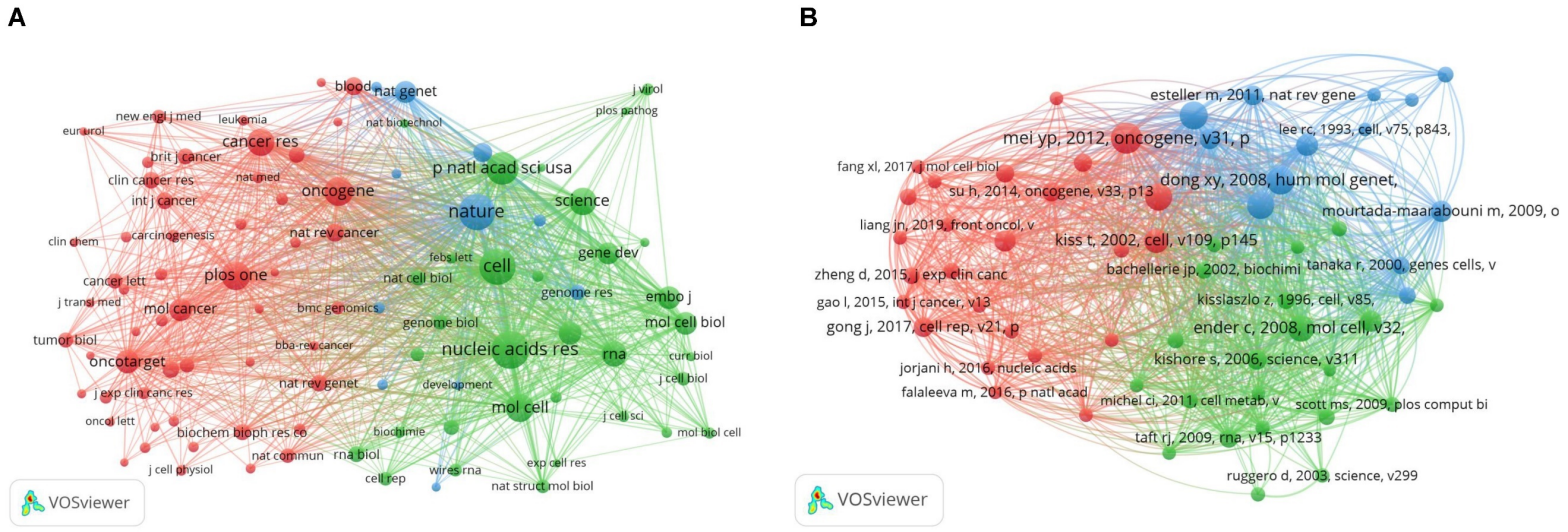
** A bibliometric analysis of co-citation in the field of snoRNA and cancer.** (**A**). The network visualization map of co-cited journals in the field of snoRNA and cancer. (**B**). The network visualization map of co-cited references in the field of snoRNA and cancer.

The main purpose of co-citation analysis was to identify the highly cited papers in the research field and the journals that published these papers. We analyzed the characteristics of journals with high citation frequency and used VOSviewer to screen journals with citation frequency of > 80. The results of visual analysis are presented **in Figure 8A**. The three most frequently cited journals were Nature, Cell, and Nucleic Acids Research, all of which were among the top journals in the natural science. These highly cited journals were divided into three clusters: the red cluster on the left (cluster 1) included journals focused on cancer research; the green cluster on the right (cluster 2) focused on biochemical molecules; and the blue cluster in the middle (cluster 3) focused on genes.

Subsequently, we carried out a visual analysis of the papers with high co-citation frequency (**Figure 8B**). In this figure, papers with high citation frequency were classified into three categories. Cluster 1 (red) comprised mainly of papers on the correlation of snoRNA with cancer, which can help us quickly understand the correlation; Cluster 2 (green) was largely comprised of articles discussing the function of snoRNAs; Cluster 3 (blue) included the relatively early articles on tumors. These articles discussed the relationship between the occurrence and development of tumor and some biomolecules, including snoRNAs. Most of them were groundbreaking articles in the field.

Subsequently, we analyzed the highly cited papers and sorted the top 10 papers based on the citation frequency (**Table 5**). It was clear that these articles laid the foundation for this research field. Reading these articles can help researchers quickly understand the background and the theoretical basis of the association of snoRNAs with cancer.

## 4. Hotspots and frontiers

Based on the above bibliometric analysis, especially the analysis of burstiness words, we summarized the hot spots and frontiers of the research on the correlation between snoRNAs and cancer and put forward the prospects.

### 4.1. Molecular mechanism of cancer induced by snoRNA

SnoRNAs are a type of non-coding RNA which are 60-300 nucleotides in length and can be classified into two types: C/D box snoRNAs and H/ACA box snoRNAs 4, 5, 27. SnoRNAs were initially believed to be involved in the processing and synthesis of rRNA 7. However, subsequent research revealed that these are also involved in the modification of transfer RNA, and small nuclear RNA 28. Since snoRNA is involved in modifying several small molecules key to transcription and translation, its significance for cell regulation was quickly recognized by researchers, and some researchers found a potential link between snoRNAs and cancer. The first study on the role of snoRNAs in cancer was conducted in the context of B-cell lymphoma, which indicated that chromosomal translocation t (3;6) (q21; q15) was associated with B-cell lymphoma. The host gene of C/D box snoRNA U50 was located at the break point of this translocation 29.

Many subsequent studies have found that U50 is also likely to act as a tumor suppressor. Downregulation of U50 expression has been demonstrated in a variety of cancers, such as prostate cancer, breast cancer, and colorectal cancer 30-32. In 2002, Chang et al. 33 reported that the expression of a snoRNA called h5sn2 was significantly lower in meningiomas than in normal brain tissue. A subsequent study also demonstrated the upregulation of snoRNAs in six non-small cell lung cancer (NSCLC) specimens, three of which were located in areas of potential oncogene amplification in lung cancer 19. These studies provided a foundation for confirming the correlation between snoRNA and cancer. Gong et al. 34 reported significant overexpression of some snoRNAs in more than 30 cancers. From a broader perspective, this study further demonstrated a close relation of snoRNAs with cancer, and that different snoRNAs can promote or suppress the occurrence and development of tumors.

The correlation between snoRNAs and cancer molecular pathology is not limited to the expression level of snoRNAs. Numerous studies have revealed various molecular pathways through which snoRNA may lead to tumorigenesis. Some early studies have found that snoRNA can influence cancer development by affecting the subcellular localization of telomerase RNA 35. This was also the beginning of research into the molecular mechanisms by which snoRNAs may be involved in carcinogenesis.

Subsequent studies have found that the p53 pathway is an important link between snoRNAs and cancer. P53 is a transcription factor that plays a central role in regulating cell cycle and is considered to be an important tumor suppressor 36. Overexpression of SNORA80E in NSCLC was found to be closely related to the decrease of p53. SNORA80E was found to promote cell proliferation and inhibit cell apoptosis through p53, and further establish and maintain tumor initiation cells of lung cancer 37. Su et al. 38 focused on fibrillarin (FBL), the enzyme unit of C/D box snoRNPs, which can cause tumors by suppressing p53 function. Overexpression of snoRNA in FBL and C/D box has been observed in both human breast cancer and prostate cancer, which also supported this idea 38. Another study found that SNORD17 may promote the growth of HCC cells by inhibiting the p53 pathway 39.

Many other molecular pathways have also been shown to mediate the effect of snoRNAs on cancer development. A recent report on lung cancer suggested that SNORA71A regulates cell cycle and epithelial-mesenchymal transition through MAPK/ERK pathway 40. Another study has found that SNORD52 can promote the occurrence of HCC by binding with CDK1 to enhance the expression of proteins 41. The PI3K-AKT pathway and Wnt signaling pathway are believed to influence cancer cell metabolism by regulating the cell cycle 42. Some snoRNAs are closely related to these two signaling pathways. Knock-down of SNORA47 was found to significantly inhibit the occurrence of NSCLC by inhibiting the PI3K-AKT signaling pathway 43. SnoRNA ACA11 can activate PI3K-AKT pathway to promote cell growth, migration and invasion, and participate in the occurrence of HCC 44. Another two snoRNAs, U2_19 and SNORD1C, were found to be involved in the occurrence of HCC and colorectal cancer by activating Wnt signaling pathways, respectively 45, 46.

There is a strong body of evidence demonstrating the strong correlation between snoRNA abnormalities and the occurrence and development of cancer. However, there are still some undefined aspects of these theories. Further research is required to identify other underlying molecular mechanisms.

### 4.2. Diagnostic and prognostic application of snoRNAs in cancer

As mentioned above, certain types of snoRNA are highly expressed in a variety of cancer types 34. Application of this theory for diagnostic and prognostic purposes can be of great clinical value. SnoRNAs have been shown to be stable and measurable in serum, highlighting their suitability for use as diagnostic biomarkers for cancer 26.

Clinical studies on lung cancer have found high expression of two snoRNAs, SNORD33 and SNORD76, in the plasma of patients with NSCLC, and the high expression level was only observed in NSCLC patients 19. If applied clinically, this feature can facilitate diagnosis and avoid false-positive results 19. Increased expression of SNORA42 and SNORA71A in lung cancer was found to be associated with poor prognosis 40, 47. Another study on lung cancer implicated SNORD60 in the causation of lung adenocarcinoma and highlighted its role as a marker of poor prognosis 48. A study identified the prognostic value of HBII-239 snoRNA in T cell lymphoma, and its elevated level indicated a good prognosis 49. Another study on hematologic tumors also indicated differential expression of snoRNAs in different B-cell tumors 27. A clinical study published in 2017 found a significant association of SNORA23 with the prognosis of HCC as high expression of SNORA23 was associated with significantly shorter survival time 50. A recent article on HCC identified three snoRNAs, namely SNORA11, SNORD124, and SNORD46, which play an important role in the diagnosis and prognosis of HCC 51. In a study by Cuicui Li, high SNORD52 expression was found to be associated with poor prognosis of HCC 41. Some snoRNAs such as H/ACA box small nucleolar RNA 7B can also affect the migration and invasion of breast cancer cells; therefore, specific snoRNAs can be used as prognostic markers of breast cancer 52, 53. Other studies on gastric cancer found that snoRA42, snoRA74A, and snoRD10 may play a potential role in the causation of gastric cancer 54, while ACA47, E2, ACA10, SNORA58, HBII-316, U70, U8, and U66, SNORA21 were identified as potential prognostic indicators of gastric cancer, as elevated levels of these snoRNAs were often associated with shorter survival time 55, 56.

These studies provided evidence of the potential role of snoRNAs as diagnostic and prognostic biomarkers in the context of cancer. SnoRNAs are not currently being applied for clinical use. Further research is required to enable the clinical use of snoRNAs as biomarkers for improving the diagnosis and prognostic assessment of these patients.

### 4.3. Potential therapeutic value of snoRNAs for cancer

Owing to the several potential mechanisms of the involvement of snoRNAs in carcinogenesis, many researchers are eager to explore its potential as a target for cancer treatment. As mentioned above, knocking down SNORA42 can prevent NSCLC to some extent, and may be an effective therapeutic target 43.

Therefore, role of snoRNAs as a therapeutic target for cancer is a promising research direction and may become a hotspot of this field in the future. Although much of the current research on treatment is still speculative, snoRNAs have shown potential value in cancer diagnosis and prognosis.

## 5. Conclusion

The role of snoRNAs in cancer is an emerging research field. Based on the analysis of the literature of the last 20 years by VOSviewer and Citespace, this study systematically reviewed the development trend in this field. We also conducted data processing and visualization analysis of high-quality authors, productive countries, and organizations, as well as core journals and keywords in the field, and reached the following conclusions:

(1) The field of snoRNAs and cancer has produced a number of high-quality authors, becoming the backbone of the field.

(2) The core journals in this field are Oncotarget, Plos one, and International Journal of Molecular Sciences.

(3) Chinese scholars published the most papers and contributed the most in this field. Investigators from England published the most frequently cited literature, which laid the foundation of extensive research in this field.

(4) Wuhan Univ, China Med Univ, and Guangxi Med Univ were the institutions that published the most papers.

(5) Analysis of key words suggests that the research direction and hotspot in this field has shifted from basic theory to clinical application.

## 6. Strengths and limitations

This is the first comprehensive and systematic bibliometric analysis of snoRNAs in the field of cancer. Compared with other traditional review articles, this study performed a more objective and comprehensive analysis in terms of quantity and relevance. However, some limitations of this study should be considered. Firstly, the literature in our study was only extracted from SCIE in WOS core collection database, which inevitably affected the comprehensiveness of the literature. Nevertheless, WOS is a comprehensive and popular online database in the field of bibliometrics. Owing to very few omissions in this database, this is unlikely to affect the main results of the analysis. Secondly, most of the literature analyzed in detail was highly cited. Newly published papers may not attract enough attention because they have been published for a short time. Finally, interpretation of the data after quantitative analysis requires a thorough and comprehensive understanding of this field. In this process, an element of subjectivity cannot be ruled out.

## Figures and Tables

**Table 1:** The top 5 most active authors in the field of snoRNA and cancer.

Rank	Author	Publications	Citations	Average Citation/Publication	Representitive Article	Publication Record	IF/JCR	Core Content
1	Chu, Liang	7	233	33.29	Small Nucleolar RNAs: Insight Into Their Function in Cancer	Front Oncol. 2019 Jul 9;9:587.	5.738/Q2	Aberrant expression and mutations in specific snoRNAs were associated with tumorigenesis and metastasis.
2	Montanaro, Lorenzo	7	152	21.71	Loss of function of the tumor suppressor DKC1 perturbs p27 translation control and contributes to pituitary tumorigenesis	Cancer Res. 2010 Jul 15; 70(14): 6026-6035.	13.312/Q1	DKC1, which functions in combination with box H/ACA snoRNA, may contribute to the development or progression of cancer by reducing p27 expression.
3	Jiang, Feng	6	461	76.83	Small nucleolar RNA signatures as biomarkers for non-small-cell lung cancer	Mol Cancer. 2010; 9: 198.	41.444/Q1	SnoRNAs existed in a stable form and were reliably measurable in plasma,and can be used as a noninvasive diagnostic tool for early NSCLC.
4	Yuan, Yufeng	5	170	34	Small nucleolar RNA ACA11 promotes proliferation, migration and invasion in hepatocellular carcinoma by targeting the PI3K/AKT signaling pathway	Biomed Pharmacother. 2017 Jun;90:705-712.	7.419/Q1	ACA11 has an oncogenic role in HCC and may serve as a promising prognostic biomarker and therapeutic target.
5	Trere, Davide	5	48	9.6	SnoRNA U50 Levels Are Regulated by Cell Proliferation and rRNA Transcription	Int J Mol Sci. 2013 Jul 17;14(7):14923-35.	6.208/Q1	Demonstrate the relationship between U50 and cellular proliferation rate and ribosome biogenesis and explain the association between U50 and cancer.

**Table 2:** The top 9 main journals in the field of snoRNA and cancer.

Rank	Source	Publications	Citations	Average Citation/Publication
1	Oncotarget	14	402	28.71
2	PLoSone	12	393	32.75
3	International Journal of Molecular Sciences	12	334	27.83
4	Oncogene	11	1347	122.45
5	Nucleic Acids Research	10	303	30.3
6	RNA Biology	9	301	33.44
7	Frontiers in Oncology	9	111	12.33
8	Molecular Cancer	8	661	82.63
9	Cancers	8	198	24.75

**Table 3:** The top 5 most active countries/regions in the field of snoRNA and cancer.

Rank	Country	Publications	Citations	Average Citation/Publication
1	China	147	3259	22.17
2	USA	124	7579	61.12
3	Italy	39	1345	34.49
4	German	30	854	28.47
5	England	27	2489	92.19

**Table 4:** The top 5 most active organizations in the field of snoRNA and cancer.

Rank	Organization	Publications	Citations	Average Citation/Publication
1	Wuhan Univ	12	245	20.42
2	China Med Univ	12	216	18
3	Guangxi Med Univ	12	80	6.67
4	Univ Texas MdAnderson Canc Ctr	11	610	55.45
5	Huazhong Uni Sci & Technol	10	309	30.9

**Table 5:** The top 10 co-cited references in the field of snoRNA and cancer.

Rank	Title	Author	Year	Citations	Publication Record	IF/JCR	Core content
1	Small nucleolar RNA 42 acts as an oncogene in lung tumorigenesis	Mei, YP	2012	107	Oncogene. 2012 May 31;31(22):2794-804.	8.756/Q1	SNORA42 caused marked repression of lung cancer growth *in vitro* and *in vivo*.
2	SnoRNA U50 is a candidate tumor-suppressor gene at 6q14.3 with a mutation associated with clinically significant prostate cancer	Dong, XY	2008	91	Hum Mol Genet. 2008 Apr 1; 17(7): 1031-1042.	5.121/Q1	SnoRNA U50 might be a 6q14-15 tumor suppressor gene in prostate cancer, and can be applied to clinical prediction.
4	Small nucleolar RNA signatures as biomarkers for non-small-cell lung cancer	Liao, JP	2010	91	Mol Cancer. 2010 Jul 27;9:198.	41.444/Q1	SnoRNAs existed in a stable form and were reliably measurable in plasma,and can be used as a noninvasive diagnostic tool for early NSCLC.
3	Implication of snoRNA U50 in human breast cancer	Dong, XY	2009	85	J Genet Genomics. 2009 Aug;36(8):447-54.	5.723/Q1	SnoRNA abnormality plays a role in breast cancer development.
6	Are snoRNAs and snoRNA host genes new players in cancer?	Williams, GT	2012	84	Nat Rev Cancer. 2012 Jan 19;12(2):84-8.	69.800/Q1	SnoRNA dysfunction contribute to oncogenesis in some new ways.
5	A Human snoRNA with MicroRNA-Like Functions	Ender, C	2008	81	Mol Cell. 2008 Nov 21;32(4):519-28.	19.328/Q1	Several snornas that function like micrornas.
7	Small nucleolar RNAs: An abundant group of noncoding RNAs with diverse cellular functions	Kiss, T	2002	72	Cell. 2002 Apr 19;109(2):145-8.	66.850/Q1	Some biological functions of snoRNA and its relationship with 2 '-O-methylation and pseudouridylation are summarized.
8	The small-nucleolar RNAs commonly used for microRNA normalisation correlate with tumour pathology and prognosis	Gee, HE	2011	60	Br J Cancer. 2011 Mar 29;104(7):1168-77.	9.075/Q1	The clear dysregulation of multiple snoRNAs and their host genes in cancer suggests a novel area of research in cancer initiation and progression.
9	GAS5, a non-protein-coding RNA, controls apoptosis and is downregulated in breast cancer	Mourtada-Maarabouni, M	2009	57	Oncogene. 2009 Jan 15;28(2):195-208.	8.756/Q1	SnoRNA U50 take part in controlling oncogenesis and sensitivity to therapy in cancer.
10	Human box C/D snoRNAs with miRNA like functions: expanding the range of regulatory RNAs	Brameier, M	2011	55	Nucleic Acids Res. 2011 Jan;39(2):675-86.	19.160/Q1	Several C/D box snoRNAs give rise to gene regulatory RNAs.

## Abbreviations

snoRNA: small nucleolar RNA
rRNA: ribosomal RNA
WOS: Web of Science
SCIE: Science Citation Index Expanded
PI3K: phosphoinositide 3-kinase
NSCLC: non-small cell lung cancer
FBL: fibrillarin
HCC: hepatocellular carcinoma
